# An Online Support Group Intervention for Adolescents Living with HIV in Nigeria: A Pre-Post Test Study

**DOI:** 10.2196/12397

**Published:** 2018-11-28

**Authors:** Lisa Dulli, Kathleen Ridgeway, Catherine Packer, Kate F Plourde, Tolulope Mumuni, Tosin Idaboh, Adesola Olumide, Oladosu Ojengbede, Donna R McCarraher

**Affiliations:** 1 Department of Program Sciences and Technical Support, Health Services Research Family Health International (FHI 360) Durham, NC United States; 2 Department of Reproductive, Maternal, Newborn and Child Health Family Health International (FHI 360) Durham, NC United States; 3 Department of Program Sciences and Technical Support, Research Utilization Family Health International (FHI 360) Durham, NC United States; 4 Center for Population and Reproductive Health (CPRH) College of Medicine University of Ibadan Ibadan Nigeria; 5 Nigeria Country Office Family Health International (FHI 360) Abuja Nigeria

**Keywords:** adolescents, digital health intervention, HIV care continuum, social support

## Abstract

**Background:**

Adolescents living with HIV (ALHIVs) enrolled in HIV treatment services experience greater loss to follow-up and suboptimal adherence than other age groups. HIV-related stigma, disclosure-related issues, lack of social support, and limited HIV knowledge impede adherence to antiretroviral therapy (ART) and retention in HIV services. The 90-90-90 goals for ALHIVs will only be met through strategies targeted to meet their specific needs.

**Objectives:**

We aimed to evaluate the feasibility of implementing a social media-based intervention to improve HIV knowledge, social support, ART adherence, and retention among ALHIV aged 15-19 years on ART in Nigeria.

**Methods:**

We conducted a single-group pre-post test study from June 2017 to January 2018. We adapted an existing support group curriculum and delivered it through trained facilitators in 5 support groups by using Facebook groups. This pilot intervention included five 1-week sessions. We conducted structured interviews with participants before and after the intervention, extracted clinical data, and documented intervention implementation and participation. In-depth interviews were conducted with a subset of participants at study completion. Quantitative data from structured interviews and group participation data were summarized descriptively, and qualitative data were coded and summarized.

**Results:**

A total of 41 ALHIV enrolled in the study. At baseline, 93% of participants reported existing phone access; 65% used the internet, and 64% were Facebook users. In addition, 37 participants completed the 5-session intervention, 32 actively posted comments in at least one session online, and at least half commented in each of the 5 sessions. Facilitators delivered most sessions as intended and on-time. Participants were enthusiastic about the intervention. Aspects of the intervention liked most by participants included interacting with other ALHIVs; learning about HIV; and sharing questions, experiences, and fears. The key recommendations were to include larger support groups and encourage more group interaction. Specific recommendations on various intervention components were made to improve the intervention.

**Conclusions:**

This novel intervention was feasible to implement in a predominantly suburban and rural Nigerian setting. Social media may be leveraged to provide much-needed information and social support on platforms accessible and familiar to many people, even in resource-constrained communities. Our findings have been incorporated into the intervention, and an outcome study is underway.

**Trial Registration:**

ClinicalTrials.gov NCT03076996; https://clinicaltrials.gov/ct2/show/NCT03076996 (Archived by WebCite at http://www.webcitation.org/73oCCEBBC).

 

## Introduction

Despite important gaps in our understanding of the HIV epidemic among adolescents (age, 10-19 years), available data indicate that this age group experiences high rates of new HIV infection, has less access to treatment, and is more likely to discontinue treatment and die from HIV-related causes than other age groups [1,2]. More than 80% of adolescents living with HIV (ALHIVs) are in sub-Saharan Africa, where, despite considerable progress, treatment coverage remains challenging: By 2016, less than 50% of ALHIVs received treatment [1-3]. Between 2010 and 2016, HIV-related mortality among adolescents decreased by 5% globally and 14% in Eastern and Southern Africa; however, in Western and Central Africa, the related mortality increased by 15% [2].

In 2016, nearly 50% of HIV-related deaths among adolescents in Western and Central Africa occurred in Nigeria [2]. One contributing factor to the high mortality may be poor treatment retention among ALHIVs. A 2016 study of youth (age, 14-24 years) initiating antiretroviral therapy (ART) in Nigeria found that they were less likely to remain in treatment at 12 months (46% vs 53%) and more likely to experience treatment disruptions (>3 months between clinic visits) in their first year on ART than older adults (relative risk=1.15, *P*=.008) [4].

Fear of stigma or disclosure, lack of social support, and limited knowledge about the disease, compounded by the physical, social, and psychological changes of adolescence, create numerous challenges to successful treatment [5-10]. For example, adolescence is a time when relationships outside of the immediate family, such as those with peers, become very important [11]; however, because of HIV-related stigma and discrimination, ALHIVs rarely disclose their status to their peers for fear of losing them, which can negatively impact their psychosocial well-being and treatment [12-13].

Evidence of effective interventions to improve treatment outcomes among ALHIVs, particularly in low- and middle-income countries, is limited [14-15]. ALHIVs are also less likely than adults or younger children to be targeted with interventions to improve health outcomes [14,15]; however, some intervention strategies designed for adults may be effective for ALHIVs. Emerging evidence supports group counseling and structured support groups as effective ways to improve adherence and retention among adults [16-21]. Digital health interventions such as mobile phone reminders improved adherence among adults in some low- and middle-income countries [22-27]. Two recent studies targeting youth in South Africa and the United States demonstrated that online social network interventions could be acceptable and feasible in this population [28-29].

According to a 2014 survey, 89% of Nigerians aged ≥ 18 years owned a mobile phone [30]. A 2012 study in Nigeria found that more than half of the females aged 12-30 years owned a phone, and almost all who did not own a mobile phone had access to one [31]. The rapid increase in mobile phone use in Nigeria indicates the potential of digital health strategies to help meet the support needs of ALHIVs.

In this study, we aimed to develop and test the feasibility and acceptability of a structured support group intervention—SMART (Social Media to promote Adherence and Retention in Treatment) Connections—which is delivered through a social media platform to improve retention in HIV health services and ART adherence among ALHIVs aged 15-19 years in periurban southern Nigeria.

## Methods

### Design

We conducted a mixed-methods, single-group, pre-post test study from June 2017 to January 2018. Participants were recruited from 3 health facilities in Akwa Ibom State and enrolled to receive 5 intervention sessions [32]. Data were collected through face-to-face interviews using a structured questionnaire at baseline and endpoint. In-depth interviews (IDIs) with a subset of purposively selected participants were conducted at endpoint and stratified by participation level. Participation data were collected using a Facebook group analytics tool [33]. The FHI 360 Protection of Human Subjects Committee, Durham, North Carolina, and the University of Uyo Teaching Hospital Ethics Committee Uyo, Akwa Ibom State, Nigeria, approved this study. Written informed consent was obtained from adult participants aged ≥18 years. Written parental permission and adolescent assent were obtained from participants aged 15-17 years.

### SMART Connections: Development and Description

SMART Connections was designed to promote ART adherence and retention in HIV services by leveraging informational, emotional, and network dimensions of social support. Content was adapted from sessions of the Positive Connections: Leading Information and Support Groups for Adolescents Living with HIV guide, to be delivered online through “secret” Facebook groups [34]. Secret Facebook groups limit membership and access to those invited and added by a group administrator. Content in secret Facebook groups can be seen only by members. These groups cannot be found through online search engines and do not appear on Facebook users’ timelines; therefore, others who are not part of the group cannot determine whether a person is a member of a secret group [35].

**Figure 1:**
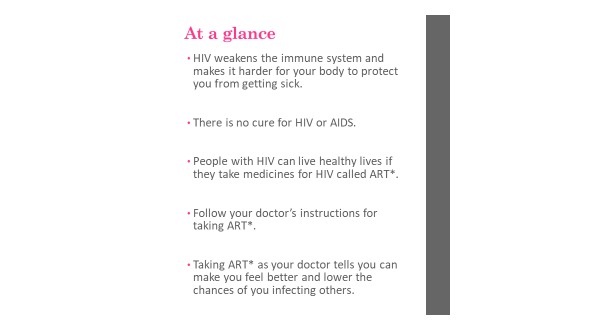
At a glance. *ART: antiretroviral therapy.

**Figure 2:**
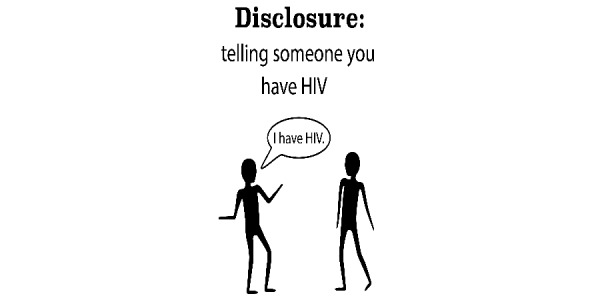
Word of the week.

**Figure 3:**
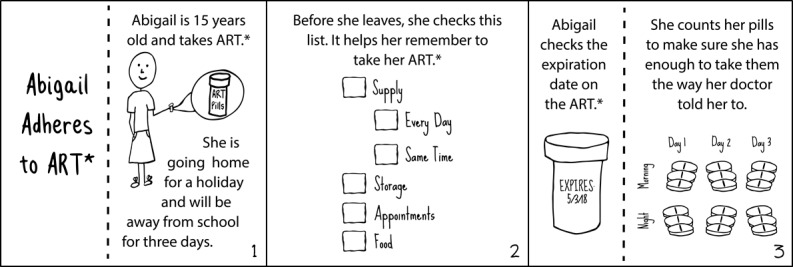
Sample cartoon. *ART: antiretroviral therapy.

Intervention components included informational messages and moderated group discussions, and 5 of 14 Positive Connections sessions were included in this study: Understanding HIV; Disclosure and Developing Trust in Relationships; Treatment and Adherence; Nutrition and Health; and Sex and Relationships. Sessions included activities for the facilitators to post and subsequently lead discussions. Within each session, the activities included the following: At a glance, to introduce the topic of the week (Figure 1); word of the week, to define one key concept for the topic (Figure 2); cartoons, to tell a story related to the topic (Figure 3); key messages, to deliver important information; quizzes, to assess participants’ knowledge and stimulate discussion; and group discussions moderated by facilitators. Four facilitators, recruited from existing community-based organizations and previously trained to lead in-person support groups for people living with HIV, received a 1-week training on the intervention.

Study support groups began with an initial, in-person meeting during which participants met each other and the facilitator. The facilitator described the intervention and the group agreed upon ground rules for interactions, emphasizing the need to maintain confidentiality for group interactions. All participants received a basic mobile phone (also known as a feature phone) that could access Facebook, regardless of current phone ownership, because our primary interest was to determine whether the intervention could be implemented as designed and if participants would engage in it, given the opportunity. Participants selected the cellular network on which the phone was registered and were allowed to keep the phones at the end of the study. Each participant received 1000 Naira (USD 3.33) of data each month to facilitate participation, corresponding to 1.5-2 gigabytes of data monthly, depending on the network provider. This amount of data translates roughly to 300-400 social media posts with photos [36].

### Sample Size and Sampling Design

We aimed to enroll 40-50 ALHIVs to form 5 support groups of 8-10 individuals. Eligibility criteria for ALHIVs for inclusion in the study were age, 15-19 years; on ART for >6 months; and basic literacy to participate in online chats. Literacy was assessed by data collectors during eligibility screening by asking the participant to read aloud three short sentences from the intervention content. If the data collector determined that the participant could read all or most of the sentences, the person was deemed eligible. Participants who could not read at all or struggled with all three sentences were deemed ineligible. ALHIVs who planned to move from the area before the end of the study, were enrolled in an in-person support group, were enrolled in another HIV-related research study, or were critically or severely ill at enrollment were excluded from the study.

Eligible participants were sequentially recruited during clinic visits. Additionally, participants were identified from medical records by a clinic staff and contacted by telephone to tell them about the study and, if interested, to come to the facility to learn more about the study. IDIs were conducted for 8 participants with moderate to high active participation and 8 participants with no to low active participation at the study endpoint.

### Measures

We collected demographic information of participants (sex, age, relationship status, education, occupation, and religion) and their HIV infection (date of diagnosis, date of start of ART, disclosure to others, viral load, and CD4 [cluster of differentiation 4] results). We also collected data on preintervention access to mobile phones, the internet, and experience with social media.

We measured intervention implementation and participation using Grytics software [33]. To measure fidelity, we determined if intervention activities occurred within 1 week of when they were scheduled and the proportion of scheduled activities completed. In addition, we measured if and how participants participated in each scheduled activity. Group members could participate in several ways: commenting on a scheduled post, “liking” or “reacting” to a post or comment, making a new post, or commenting on or reacting to others’ posts. IDIs explored participants’ experiences with the intervention.

### Data Analysis

Quantitative data were analyzed descriptively and independently verified by a second analyst. For participation data, posts (original message) and comments (replies to a post) were equally weighted and each assigned a value of 1. Participation was categorized into approximate quartiles of total posts over the 5 sessions: none to very low (0-5 total posts), low (6-20 posts), moderate (21-50 posts), and high (≥51 posts).

IDIs were audio recorded and transcribed into English. Some IDIs were conducted in local languages and then simultaneously transcribed and translated. Qualitative data were analyzed by an applied thematic approach using NVivo 11 [37,38]. A codebook was created on the basis of the interview guide, and emergent thematic codes were added during analysis. Two analysts coded the transcripts and checked 12% of the transcripts for intercoder reliability. Summary memos documented overall themes related to the study objectives.

## Results

**Figure 4:**
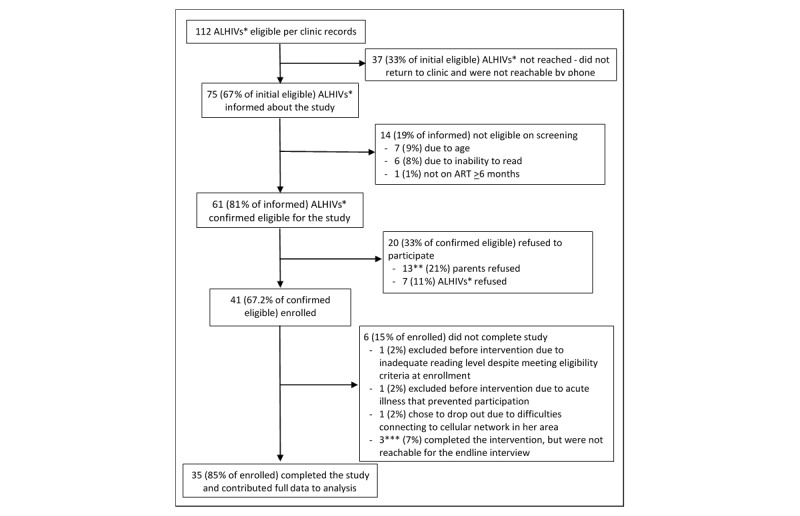
Study flow chart. *ALHIV: adolescent living with HIV. **In 5 cases, the guardians refused to let the child participate because they had not yet disclosed their child's HIV status to him or her. ***One participant did not complete the endpoint structured interview, but completed the in-depth interview.

A total of 41 adolescents enrolled in the study and completed a baseline interview, of whom 38 started the intervention and 35 completed the endpoint questionnaire. A flow chart describing the identification, enrollment, and follow-up of participants is presented in Figure 4.

Several individuals initially deemed eligible according to clinical records were later found to be ineligible due to incorrect age information in their medical records. Of those confirmed to meet eligibility criteria, 13 parents or guardians refused to allow their child to participate, 5 of whom stated they had not yet disclosed their child’s HIV status to him or her.

Participants were nearly equally divided by sex (Table 1). About half of the participants were enrolled in school, mostly in secondary school, and one-quarter were employed. All participants had a home or primary residence. Most participants had been on ART for >4 years; 90% (37 of 41) had at least one CD4 cell count test recorded in their medical record, but only 73% (30 of 41) had a viral load test recorded in their medical record (Table 1). Among those with a recorded viral load, half were virally suppressed (Table 1). All participants who were not virally suppressed had viral loads >5000 copies/mL (data not shown).

**Table 1:** Participant characteristics at baseline.

Characteristic (N=41)		Value
**Sex, n (%)**		
	Female	22 (53)
	Male	19 (46)
Age (years), median (range)		17 (15-19)
Currently employed, n (%)		11 (26)
**Education, n (%)**		
	Currently enrolled in secondary school	18 (43)
	Currently enrolled in postsecondary school	2 (4)
	Not currently enrolled, completed secondary school	15 (36)
	Not currently enrolled, completed some secondary school	6 (14)
**Among those in school, location of residence during school (n=20), n (%)**		
	Live at home	19 (95)
	Boarding school	0 (0)
	With a friend’s family	1 (5)
Has a primary household, n (%)		41 (100)
**Religion, n (%)**		
	Christian denomination	41 (100)
Time on antiretroviral therapy^a^ (years), median (range)		4.0 (0.5-11.0)
CD4^b^ cell count at last test (cells/µl)^c^, median (range)		414 (16-957)
**Viral load at last test^d^, n (%)**		
	Suppressed viral load (<1000 copies/mL)	15 (50)
	Unsuppressed (≥1000 copies/mL)	15 (50)
	Viral load (copies/mL), median (range)	31,210 (5338-936,973)

^a^Data for 6 participants missing.

^b^CD4: cluster of differentiation 4.

^c^Data for 4 participants missing.

^d^Data for 11 participants missing.

Three-quarters of all participants lived with one or both parents, and the rest lived with other family members, including one participant who was married and lived with the spouse (Table 2). The majority (68%) reported that at least one parent knew of their HIV status; fewer participants had other family members who knew their status, and only 3 (7%) reported that a close friend knew their status. Six participants stated they were in romantic relationships, and all but one participant reported that their partner knew their HIV status; 5 of the 6 participants knew their partner’s HIV status.

### Feasibility

At baseline, nearly all participants (93%) had existing access to a phone, and most (66%) ever used the internet (Table 3). Nearly all participants (96%) who used the internet had used Facebook. Among those who had phone access before this study, most reported difficulty charging phones (79%) or running out of airtime or phone credit (97%) sometimes or often.

Nearly all participants (92%) who completed the endpoint structured interview reported that connecting to Facebook was somewhat or very easy, and 97% stated that they wrote comments or asked questions during sessions (Table 4).

#### Participant Engagement

Most participants who completed the endpoint questionnaire (34 of 35) participated in the intervention sessions (Table 5). The majority of intervention participants took part in all sessions and reported actively commenting or asking questions at some point.

**Figure 5:**
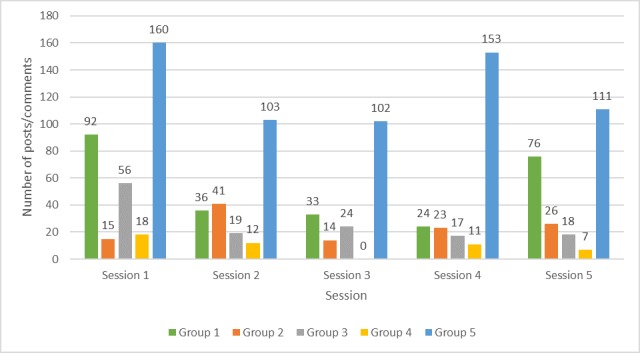
Group posts per session.

**Figure 6:**
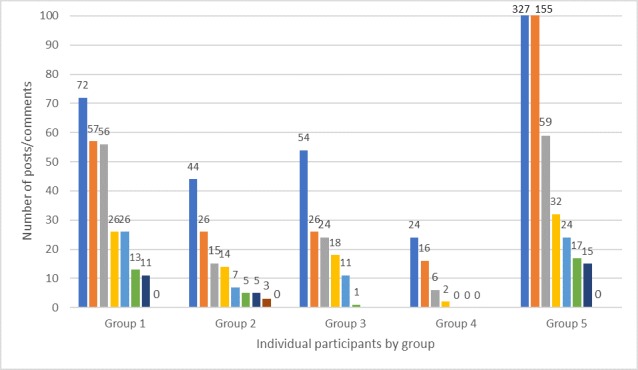
Individual posts per group.

Participation varied widely by session and group (Figure 5). Within groups, participation varied considerably (Figure 6). Each group had 1-3 members who were considerably more active than the others. Two participants made >100 posts (327 and 155 posts; Figure 6).

**Table 2:** Relationships and disclosure status at baseline.

Characteristic (N=41)		n (%)
**Lives with**		
	Both parents	14 (34)
	Mother only	12 (29)
	Father only	5 (12)
	Spouse or partner	1 (2)
	Other relative (sister, grandparents, cousin, aunt, or uncle)	9 (21)
**Others who know the participant’s HIV status^a^**		
	Parent(s)	28 (68)
	Sibling(s)	12 (29)
	Other family member(s)	14 (34)
	Close friend(s)	3 (7)
	Religious leader	3 (7)
	No one	3 (7)
**Relationship status^b^**		
	Married	1 (2)
	Unmarried, in a relationship	5 (12)
	Single	34 (85)
Spouse, girlfriend, or boyfriend knows the participant’s HIV status (n=6)^c^		5 (83)
**Spouse, girlfriend, or boyfriend has HIV (n=6)**		
	Yes	2 (33)
	No	3 (50)
	Don’t know	1 (16)

^a^More than one response possible.

^b^Data for one participant missing.

^c^One person reported that no one knew her status besides the health providers, but then reported that her partner knew her status.

**Table 3:** Mobile phone and internet use from the baseline questionnaire.

Characteristic (N=41)			n (%)
**Mobile phone access**			
	None		3 (7)
	Has own basic phone		16 (39)
	Has own smart phone		3 (7)
	No personal phone but has access to phone in household		19 (46)
**Among those who own or have access to phone (n=38)**			
	**Ways of typically using the phone^a^**		
		Make voice calls	38 (100)
		Send texts or SMS^b^	34 (91)
		Send group texts or MMS	12 (31)
		Access internet	21 (55)
		Access social media	22 (57)
	**Frequency of running out of airtime or credit**		
		Rarely	1 (2)
		Sometimes	27 (71)
		Often	10 (26)
	**Frequency of facing difficulty charging phone**		
		Never or Rarely	8 (21)
		Sometimes	25 (65)
		Often	5 (13)
Ever used the internet			27 (65)
**Among those who ever used the internet (n=27)**			
	**Source of internet access^a^**		
		Own computer or laptop	0 (0)
		Computer or laptop in household	2 (7)
		Computer or laptop at friend’s house	5 (18)
		Cyber cafe	2 (7)
		Own phone or tablet	9 (33)
		Phone or tablet in household	21 (77)
		Friend’s phone	1 (3)
	**Ever used social media sites^a^**		
		Facebook	26 (96)
		WhatsApp	12 (44)
		Instagram	3 (11)
		Other (Twitter, Palmchat, IMO)	7 (25)

^a^More than one response possible.

^b^Data for one participant missing.

**Table 4:** Intervention access reported in the endpoint questionnaire.

Parameter (N=35)		n (%)
Used the study phone to connect to Facebook		34 (97)
**Status of the study phone at endpoint**		
	In participant’s possession	33 (94)
	Stolen	2 (5)
Participated in the intervention at least once		34 (97)
**Among those who participated at least once (n=34), level of ease connecting to Facebook with the phone**		
	Very difficult	2 (5)
	Somewhat easy	11 (32)
	Very easy	21 (61)

**Table 5:** Intervention engagement in the endpoint questionnaire.

Parameter (N=34)^a^		n (%)
**Logged in at least once for a session**		
	Session 1—Understanding	29 (85)
	Session 2—Disclosure and trust^b^	20 (65)
	Session 3—Treatment and adherence^c^	25 (76)
	Session 4—Nutrition and health	29 (85)
	Session 5—Sex and relationships	30 (88)
**Ways engaged**		
	Read what the facilitator posted^c^	33 (100)
	Read comments posted by others	34 (100)
	“Liked” comments by others^d^	26 (87)
	Wrote comments or asked questions	33 (97)
**Engagement level during scheduled intervention (by number of posts or comments, n=38)^e^**		
	Very low (0-5)	11 (29)
	Low (6-20)	11 (29)
	High (21-50)	9 (24)
	Very high (51-327)	7 (18)

^a^One participant did not participate in the online group in the endpoint questionnaire and was not asked the questions in this table.

^b^Data for 3 participants missing.

^c^Data for 1 participants missing.

^d^Data for 4 participants missing.

^e^All participants who started the intervention.

**Figure 7:**
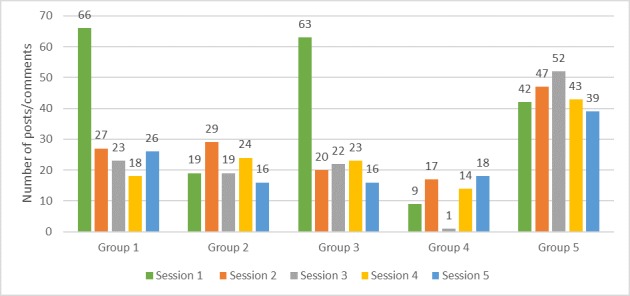
Number of posts or comments by group facilitators per session.

One group (Group 5) was the most active group overall, and the group’s facilitator was the most active among all facilitators (Figure 7). Similarly, the group with the lowest member participation had a facilitator with the lowest activity.

#### Challenges to Participation

Through the IDIs, we explored the challenges faced during implementation that might affect participation. Just over half of the 16 IDI respondents mentioned occasional problems with charging their phone or running out of data. About one-third of IDI respondents mentioned issues with cellular networks, including poor connectivity or slow data speed, of whom 2 participants lacked network coverage when they traveled and 3 had persistent network problems at home. One of them commented the following:

Whenever I don’t have data I can’t use the internet...when you are chatting online, like you are chatting somehow it can cut off.

low engager, 15-year-old male

During the session on adherence, participants were asked to upload a photo of an adherence plan; however, none of the participants did so. Several IDI respondents described difficulties with photo uploads as the reason for not uploading an adherence plan photo. One other IDI respondent noted that the pictures did not always display correctly on his phone.

One IDI respondent said he thought another group member was sharing information publicly from the Facebook group, although he did not state that his HIV status or that of others was compromised:

Some of our members...will just pick what supposed to be post in [group name] to public Facebook and everybody will see it. I used to see some of them, some people who are in the public group start asking questions, are you, where, who are, where are from...So sometimes I used to sit them down in the Facebook and warn them.

high engager, 18-year-old male

#### Fidelity to Design

Overall, each of the facilitators for the 5 groups posted most of the scheduled activities and did so on time (Table 6). Quizzes (polls) aimed to assess knowledge and stimulate discussion proved problematic. IDI respondents noted that quizzes did not appear correctly formatted on their phones. In response, facilitators posted quiz questions and response options as comments.

**Table 6:** Proportion of activities posted and posted on time per session.

Parameter		Session 1 (6 activities), %	Session 2 (7 activities), %	Session 3 (7 activities), %	Session 4 (7 activities), %	Session 5 (7 activities), %	All sessions, %
**Proportion of all activities posted (regardless of timing)**							
	Group 1	100	100	100	71	100	94
	Group 2	100	100	86	100	100	97
	Group 3	100	100	100	100	86	97
	Group 4	83	86	14	43	71	60
	Group 5	100	100	100	100	85	97
	All groups	97	97	80	83	88	
**Proportion of scheduled activities posted on time (within 1 week of schedule)**							
	Group 1	100	100	100	71	100	94
	Group 2	100	100	86	100	100	97
	Group 3	100	100	100	100	86	97
	Group 4	83	57	0	43	71	51
	Group 5	100	100	100	43	0	69
	All groups	97	91	77	71	71	N/A^a^

^a^N/A: not applicable.

#### Intervention Acceptability

All participants who completed the endpoint questionnaire agreed that the intervention was useful: They enjoyed taking part in the Facebook group, felt comfortable with the facilitator and other group members, and wanted to continue to participate in the group (Table 7). In addition, participants unanimously felt that the Facebook group intervention was a good way for ALHIVs to interact and would recommend the group to other ALHIVs.

When asked to describe what they liked about the intervention, IDI respondents, both low and high engagers, most commonly reported that they enjoyed the intervention because it was educational or informative. They enjoyed “chatting,” sharing experiences, and communicating with other ALHIVs; it helped them take care of themselves and their health (eg, most commonly, taking drugs on time) and felt supported or encouraged:

I am a very timid and shy person. But the intervention helped me. There are certain things I was able to overcome. I felt so miserable when I found out that I’m positive but after interacting with people, I find out that I don’t have to kill myself or die or feel miserable...I have decided to open up and feel good about myself.

low engager, 18-year-old female

It has affected my life in a way that, I don’t have anything like stigmatization in me I don’t and I don’t feel someone can discriminate me then I will start looking down on myself. I feel comfortable, I feel I have confidence in myself. Like disclosure, I’m not afraid, am not shame, I’m not shy to expose everything to my partner when is time to get married. So, I really like it has really done a lot of things in my life.

high engager, 18-year-old male

A few participants mentioned that they liked the confidentiality of the online forum and learning how to use Facebook:

I feel safe from exposing my status to other people. It was very good, I like it…Well, what I feel about having a Facebook group is very, very secured.

high engager, 18-year-old male

One participant was concerned about people learning of her HIV status by looking at the phone, but now feels comfortable:

At first, I was very scared. I was like what if someone should just carry my phone and see and say ha! what is this? But I was scared,…later on I became used to it, I wasn’t afraid if someone should pick up my phone and see it.

high engager, 18-year-old female

A few participants said they did not like low participation from other group members:

There’s something I didn’t like because we were 8 in number in that group and anytime or sometimes when I go online, I will only see only one chat or sometimes I won’t see anybody.

high engager, 16-year-old male

### Future Directions

Participants had few specific recommendations to change the intervention. Recommendations from the endpoint questionnaires (Table 7, n=34) included the following: encourage group members to be more active (23%); increase the size of the group (14%); provide better instructions for Facebook (8%); and use of other social media, specifically WhatsApp (5%). Two participants thought some group members may require more explanation on using Facebook in terms of etiquette for posting comments. When asked whether both sexes should be included in future groups, 94% of endpoint questionnaire respondents said yes (Table 7). Just over half the participants (55%) favored inclusion of older youths, but several wanted to cap the age at 19 years. Participants who wanted to include older youths said younger participants could learn from older participants; those who preferred the current age range said younger ALHIVs may be afraid or less open to interacting with older youths. When asked whether they would prefer to be part of an online support group, in-person support group, or both, 58% of participants preferred an online group only and 41% preferred both groups. None of the participants preferred an in-person support group without the online group.

**Table 7:** Participants’ perspectives on the intervention in the endpoint questionnaire.

Characteristic (N=34)^a^				n (%)
**Agree with the following**				
	Enjoyed being a member of the online support group			34 (100)
	Received useful information			34 (100)
	Participating in the group improved understanding of HIV			34 (100)
	Felt comfortable interacting with other HIV-positive young people in the group			34 (100)
	Felt comfortable interacting with the group facilitator			34 (100)
	Made new friends in the group			30 (88)
	Would like to continue to be part of the group			34 (100)
	Thinks Facebook groups are a good way for ALHIV^b^ to interact			34 (100)
	Thinks Facebook groups are a good way for support group leaders to get information to ALHIVs			34 (100)
	Would recommend this group to other young people living with HIV			34 (100)
**Ways to improve the intervention**				
		Encourage participation		8 (23)
		Increase group size		5 (14)
		Improve participant knowledge on Facebook use		3 (8)
		Include WhatsApp texting		2 (5)
		Specify a time to log in and be active		1 (2)
		More encouragement for participants to ask questions		1 (2)
		Encourage phone calls between participants		1 (2)
		Have a monthly group meeting		1 (2)
		Introduce the intervention elsewhere so others can benefit		1 (2)
**Recommendations for future group structure**				
	Groups should remain mixed sex			32 (94)
	Groups should include older youths (aged up to 21 or 22 years)			19 (55)
	**Would prefer support group that met**			
			Online only	20 (58)
			In-person only	0 (0)
			Combined online and in-person	14 (41)

^a^In the endpoint questionnaire, one participant said they did not participate in the online group and was not asked the questions in this table.

^b^ALHIV: adolescents living with HIV

## Discussion

This online support-group intervention was feasible to implement and highly acceptable among the ALHIVs who participated in the study. We obtained important information on specific challenges and ways to improve the intervention in order to enhance delivery and participation.

The level of active participation varied, within and across groups, which is similar to in-person support groups [21]. Group participation appeared to correlate with facilitator activity, although other factors such as logistic issues also played a role in individual participation within groups. Notably, the facilitator with the lowest overall participation performed the worst on timely and complete posting of sessions. In the future, experienced, dynamic facilitators with adequate mastery of intervention delivery should be recruited; however, even participants with low active participation reported high levels of satisfaction and appear to have benefitted from the intervention. With social media, the ability to follow conversations may allow those who are not comfortable commenting, to learn from others in the group [39,40]. Basic literacy was an important factor to the success of the intervention. Education levels are relatively high in southern Nigeria; 75.9% of women aged 15-49 years have completed some secondary school or higher education [41]. As such, implementing this intervention for individuals with low education levels could prove more difficult.

The time taken for identification and enrolment of adolescents through health facilities was longer than anticipated. Although Nigeria bears the second-highest burden of HIV on the continent in terms of absolute numbers of people infected [42], its prevalence is relatively low, with higher pockets of concentration in certain geographic areas [43]. According to the recently conducted AIDS Indicator Survey in Akwa Ibom State (2017), HIV prevalence among adolescents aged 15-19 years is 1.5% [44]. Facility staff attempted, without success, to reach several patients through their recorded contact information, which highlights the challenge of poor retention in health care services among this age group. Although inclusion of fewer eligible participants than anticipated complicated study enrollment, it supports the potential role of an online intervention that does not require individuals to live near each other or travel to a designated location and could connect people across a broader geographic area.

We also faced a few challenges during implementation of the study. Considering the limitations of the study phones, future interventions should use smartphones, which are increasingly popular and affordable, even in low- and middle-income countries [45]. Network coverage problems also limited use of the intervention among some participants. Despite these challenges, most subjects participated in most sessions.

Surprisingly, all participants who voiced an opinion preferred online support groups, either alone or in combination with an in-person group, but none of them preferred in-person groups alone. The global trend toward use of and comfort with social media among young people seems to have reached youths in periurban and rural southern Nigeria [46].

This study had a few limitations. The nonprobability sample prevents generalization of the results beyond the study sample. Social desirability bias may play a role in participants’ responses to questions about the intervention. In addition, we adapted only 5 of 14 sessions from Positive Connections; therefore, we do not know if participation may change with a longer curriculum. Moreover, we could not determine if facilitators drove participation or group member participation drove facilitator engagement.

In conclusion, this feasibility study demonstrated that an online support group intervention was both feasible and acceptable among ALHIVs in southern Nigeria. Our results provide guidance on changes required to enhance participation. Based on these findings, we have adapted the intervention and expanded it to include the remaining Positive Connections topics. A subsequent randomized controlled trial will test the cost-effectiveness of this revised intervention to improve HIV treatment outcomes.

## Abbreviations

ALHIV: Adolescents living with HIV
ART: antiretroviral therapy
CD4: cluster of differentiation 4
IDI: In-depth interview
